# Velopharyngeal Insufficiency Treatment in Cleft Palate Patients: Umbrella Review

**DOI:** 10.3390/biomimetics7030118

**Published:** 2022-08-26

**Authors:** Francisco Vale, Anabela Baptista Paula, Raquel Travassos, Catarina Nunes, Madalena Prata Ribeiro, Filipa Marques, Flávia Pereira, Eunice Carrilho, Carlos Miguel Marto, Inês Francisco

**Affiliations:** 1 Institute of Orthodontics, Faculty of Medicine, University of Coimbra, 3000-075 Coimbra, Portugal; 2 Coimbra Institute for Clinical and Biomedical Research (iCBR), Area of Environment Genetics and Oncobiology (CIMAGO), Faculty of Medicine, University of Coimbra, 3000-075 Coimbra, Portugal; 3 Centre for Innovative Biomedicine and Biotechnology (CIBB), University of Coimbra, 3000-075 Coimbra, Portugal; 4 Clinical Academic Center of Coimbra (CACC), 3030-370 Coimbra, Portugal; 5 Institute of Integrated Clinical Practice, Faculty of Medicine, University of Coimbra, 3004-531 Coimbra, Portugal; 6 Institute of Experimental Pathology, Faculty of Medicine, University of Coimbra, 3004-531 Coimbra, Portugal

**Keywords:** cleft palate, velopharyngeal insufficiency, palatopharyngeal, systematic review

## Abstract

Velopharyngeal insufficiency may occur as a result of an anatomical or structural defect and may be present in patients with cleft lip and palate. The treatment options presented in the literature are varied, covering invasive and non-invasive methods. However, although these approaches have been employed and their outcomes reviewed, no conclusions have been made about which approach is the gold-standard. This umbrella review aimed to synthesize the current literature regarding velopharyngeal insufficiency treatments in cleft lip and palate patients, evaluating their effectiveness based on systematic reviews. A standardized search was carried out in several electronic databases, namely PubMed via Medline, Web of Science, Cochrane Library, and Embase. The quality of the included studies was evaluated using AMSTAR2 and degree of overlap was analyzed using Corrected Covered Area. Thirteen articles were included in the qualitative review, with only 1 in the non-invasive method category, and 12 in the invasive method category. All reviewed articles were judged to be of low quality. In symptomatic patients, treatment did not solely comprise speech therapy, as surgical intervention was often necessary. Although there was no surgical technique considered to be the gold standard for the correction of velopharyngeal insufficiency, the Furlow Z-plasty technique and minimal incision palatopharyngoplasty were the best among reported techniques.

## 1. Introduction

Cleft lip and palate (CLP) are one of the most common congenital anomalies with a global prevalence of 1:700 [1]. This condition is characterized by the lack of fusion in facial structures, which usually occurs between the 5th and 10th weeks of pregnancy [2]. There is no single cause for this congenital anomaly, and it is thought to have multifactorial etiology [2,3]. Some risky behaviors during pregnancy are known to predispose the fetus to this condition, such as alcohol and tobacco consumption, anti-epileptic drugs or corticosteroids, and inadequate nutrition [2,3]. Additionally, people of low socioeconomic status have been reported to have a higher prevalence of orofacial clefts [2].

After primary bone graft surgery, around 20 to 50% of patients develop velopharyngeal insufficiency (VPI) [4]. Some factors are considered to increase risk for VPI, such as being of the male sex and having a shorter palate length or a wider cleft [5].

VPI can be described as the insufficient closure between the soft palate and the posterior wall of the pharynx [6,7,8]. This condition occurs in open cleft palates, submucosa, or CLPs that were surgically closed, but remained low or immobile due to the remaining scar tissue or irregular palate muscle positioning [6,9]. The main methods used to diagnose VPI are through auditory-perceptive evaluations and video naso-endoscopies [8].

In regular circumstances, the velopharyngeal valve is made up of lateral and posterior pharyngeal walls and the soft palate. When this valve closes, it divides the oral cavity and the nasal cavity. This can be observed in many instances, such as when an individual is swallowing, speaking, or breathing [9]. However, in CLP patients that present palatal muscle debilitation, there is no velopharyngeal closure during phonation, which leads to air and acoustic energy being emitted by the nasal cavity [5,10]. This results in a hypernasal speech pattern, nasal air emission, compensatory articulation, and nasal regurgitation [5,7,8,9,10].

The initial approach for VPI treatment involves speech therapy. Nonetheless, open surgery may be required in certain cases, such as cases with structural issues, a subpar speech therapy response, serious VPI, a low palate, or inadequate mobility [7,9,10]. Speech therapy can be used in both pre- and post-surgical stages. However, when it comes to VPI surgery, there is no predetermined timing nor a standardized surgical protocol [9,10]. The most common surgical procedures for VPI treatment include a pharyngeal graft or sphincter pharyngoplasty, which can reach a normal resonance of 76 and 61%, respectively [3,4,9]. Although both of these techniques are highly effective, they also present a risk of developing obstructive sleep apnea (OSA), which ranges from 19 to 93% [3,4,11]. Taking this into account, palatoplasty has emerged as an alternative with a lower risk for developing OSA [3,4]. This procedure is performed with a straight incision into the palatal mucosa or a double-opposing Z-plasty, also known as a Furlow palatoplasty [4]. Despite having an 82% success rate and not compromising the upper airway, this technique is not broadly used [3,4]. In patients with moderate VPI, autologous or synthetic implantable materials, such as cartilage, fat, or silicone, have been used to augment the posterior pharynx wall [9,10,11]. Besides the conventional surgical treatments for VPI in patients with CLP, there are also prosthetic options, such as speech bulbs or palatal lifts, that can aid in velopharyngeal closure [12].

The current literature comprises several systematic reviews that attempt to homogenize results (which remain fickle), especially when it concerns the best surgical techniques and most appropriate timing for intervention. However, no conclusions have been made about which treatment is the gold-standard. Notwithstanding, the fundamental goal of any treatment is to restore patient loss of function and form. VPI may lead to functional problems in swallowing, speaking, and breathing. Biomimetics aim to study the phenomena and processes of nature in order to understand them, and then use and modify these mechanisms; nothing is more biomimetic than the materials and techniques used to restore normal tissue function to the patient. This umbrella review aimed to access the clinical effectiveness of VPI treatments that restore the loss of structure and function by mimicking the normal appearance, form, and function of healthy human tissue using Bioinspired, biomedical, and biomolecular tissue engineering strategies and materials. Therefore, this umbrella review aims to answer the following research question: “What are the most effective treatments for velopharyngeal insufficiency in cleft palate patients?”

## 2. Materials and Methods

### 2.1. Protocol Registration

This review was registered with the International prospective register of systematic reviews (PROSPERO) under the ID number: CRD42022287414. This review was carried out in accordance with the guidelines recommended by Cochrane and PRISMA for systematic reviews.

### 2.2. PICO Question

The research question aimed to answer the clinical question: What is the most effective methodology to resolve velopharyngeal dysfunction in cleft palate patients?

The question was formulated according to the PICO principles, as described in Table 1.

### 2.3. Search Strategy

A standardized search was carried out in June 2022 in several electronic databases, such as PubMed via Medline, Web of Science, Cochrane Library, and Embase. The search keys are described in Table 2.

A search of articles in the grey literature was also carried out using the websites ProQuest (https://www.proquest.com, accessed on 20 June 2022) and OpenGrey Europe (https://opengrey.eu, accessed on 20 June 2022). Other relevant cross-references were also considered.

### 2.4. Eligibility Criteria

The inclusion criteria were established according to the PICO question above. All systematic reviews with and without meta-analyses of randomized clinical trials, non-randomized clinical trials, and case-control studies that analyzed the resolution of velopharyngeal dysfunction through various invasive and non-invasive methods were included. Only studies that evaluated the resolution of velopharyngeal dysfunction through the assessment of hypernasality were included. Studies that included literature reviews, case reports, or case series were excluded.

### 2.5. Study Selection and Data Collection

The search and studies selection were performed by two investigators (R.T. and C.N.). The studies were all selected by title or abstract according to the defined eligibility criteria, by the two researchers. In the event of disagreement, a third investigator (I.F.) assessed and resolved eligibility. After being selected for full reading and inclusion in the umbrella review, the authors extracted the following data: author and year, registration in Prospero, type of studies included in the systematic reviews, analysis of their bias and quality of evidence, age of participants, type of intervention performed, comparison group, primary outcome, and main and significant results. These results were summarized.

### 2.6. Quality Assessment

Quality assessment of the included studies was performed using the AMSTAR2 tool (Assessment of Multiple Systematic Reviews Checklists, accessed on 6 July 2022). This checklist contains several questions about the systematic reviews being evaluated to assess the quality of each one. This quality assessment was performed by two investigators (F.M. and A.P.) independently and in duplicate. Another three investigators (M.R., F.P., and C.M.M.) independently assessed the quality of the studies, and in case of disagreement with the initial evaluation, this point was discussed, and an agreement was reached by the five evaluators. The studies were classified as: high quality, in the case of zero to one weak parameters; moderate quality, in case of more than one weak parameter; and low quality, in cases where several parameters were weak.

### 2.7. Analysis of the Degree of Overlap in Studies

The analysis of the degree of overlap of selected studies between systematic reviews was performed through “Corrected Covered Area” (CCA). The overall overlap was categorized as slight (CCA = 0–5), moderate (CCA = 6–10), high (CCA = 11–15), and very high (CCA > 15).

## 3. Results

### 3.1. Study Selection

**Figure 1:**
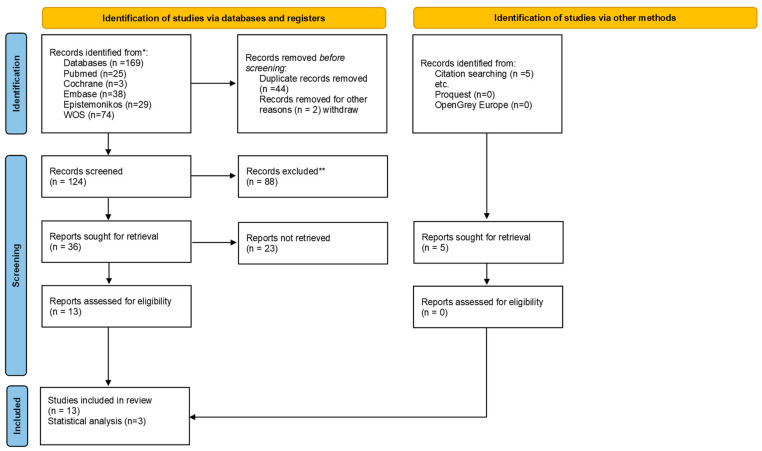
Flowchart of umbrella review.

The selection scheme (flow chart) for this umbrella review is shown in Figure 1. The initial search in the different databases resulted in 169 articles, without additional documents from the grey literature, but with 5 articles included by manual searching. After removing duplicates, 124 articles were left for screening. Reading by title and abstract resulted in 36 articles for full reading because they met the eligibility criteria. Thirteen articles were included in the final qualitative review, with only 1 in the non-invasive category and 12 in the invasive method category.

### 3.2. Description of the Included Reviews

Of the articles included in the qualitative analysis, 1 had a non-invasive method (Table 3), whereas the remaining 12 had invasive methods (Table 4). The non-invasive method was nasopharyngoscopy biofeedback, and it was evaluated in 1 systematic review. The primary evaluated outcomes were activation of lateral pharyngeal wall, velopharyngeal closure in articulation, reduction of hypernasality, nasal emission or nasal turbulence, and improvement of articulation or intelligibility in connected speech. This systematic review showed that there were no studies measuring the effectiveness of nasopharyngoscopy biofeedback without additional treatments like secondary surgery or speech-language therapy; therefore, this non-invasive method was shown to be effective only in combination with conventional speech therapy.

Invasive methods were evaluated in 9 systematic reviews and 3 systematic reviews with meta-analyses. These articles compared various interventions, namely, minimal palatopharyngoplasty with or without additional individualized velopharyngeal surgery, cleft palate repair surgical techniques, injection pharyngoplasty, pharyngeal flap surgery, and adenoidectomy. Cleft palate repair surgical techniques included Furlow double-opposing Z-plasty, straight-line intravelar veloplasty, and radical intravelar veloplasty alone or in combination with mucosal lengthening. The injection pharyngoplasty was made with autologous fat, GAX collagen, calcium hydroxyapatite, dextranomer, hyaluronic acid, and acellular dermal matrix. The resolution of velopharyngeal dysfunction was assessed in most articles through speech and hypernasality evaluation and assessing velopharyngeal gap size at rest and closure using videofluoroscopy, magnetic resonance imaging, and nasometry.

### 3.3. Quantitative Synthesis of the Results

Quantitative synthesis of the results was not possible due to heterogeneity in design and methodologies of the selected studies as well as a distinct comparison of treatments.

### 3.4. Quality of Included Reviews

Table 5 presents the quality assessments of the selected systematic reviews. Only two reviews clearly described the PICO question because most studies failed to name a comparator group. Successful registration was carried out by one review and partially in five others. Most of the included studies, except one, failed to explain their selection process. The search was not fully explained in any review. Three studies did not perform data selection and extraction of duplicates. The list of excluded studies wasn’t presented in any review. A description of selected studies was given in adequate detail in only one study, and partially done in eight other reviews. Assessing risk of bias was not performed in six reviews. No reviews reported the funding of included studies. Ten systematic reviews did not present a meta-analysis. Most reviews discuss the heterogeneity observed in the results, except for three. No studies included an assessment of publication bias. Half of the reviews reported potential conflicts of interest, including any funding they received for conducting the review. Overall, according to the AMSTAR 2 tool criteria, all reviews were considered to be of low quality.

### 3.5. Analysis of the Degree of Overlap in Studies

The 13 systematic reviews included 270 studies, of which 43 overlapped in two or more systematic reviews (Table 6). The CCA was 0.0158 (1.58% overlap). This signifies a slight overlap, meaning that a small number of studies are cited several times across the included reviews. Notwithstanding, only three of the included systematic reviews did not have overlap. Although it was expected that the most recent reviews would present a higher number of overlaps, we did not find a relationship between the year of publication and overlap number.

## 4. Discussion

This umbrella review aimed to synthesize the current literature regarding velopharyngeal insufficiency in cleft patients, evaluating their effectiveness based on systematic reviews with and without meta-analyses.

Velopharyngeal insufficiency may occur because of an anatomical or structural defect that results from incomplete closure between the soft palate and posterior pharyngeal wall, resulting in an opening between the oral and nasal cavities [17]. This pathology may be present in patients with cleft lip and palate, and is characterized by difficulty swallowing, hypernasality, and difficulty in speech articulation, which ultimately results in a lower quality of life [7,11]. In these cases, the main goal of treatment is to restore nasopharyngeal and oropharyngeal function, allowing for improved speech articulation [12]. The treatment options presented in the literature are varied, including invasive and non-invasive methods depending on the severity of the insufficiency. These include speech and swallowing therapy, prosthesis placement, palatoplasty, pharyngoplasty, muscle repositioning, and posterior pharyngeal wall enlargement procedures, such as injection augmentation pharyngoplasty (IPA) [7,12,13]. Regarding non-invasive methods, speech therapy is the most referenced method. This treatment entails long and continuous follow-up, which may contribute to exhaustion and decreased collaboration. This may explain why only one systematic review included in this study addressed speech therapy.

In symptomatic patients, treatment does not solely comprised speech therapy, as surgical intervention is often necessary [7]. Although there is no surgical technique considered to be the gold standard for the correction of velopharyngeal insufficiency, the Furlow Z-plasty technique, minimal incision palatopharyngoplasty (MIPP), and other modified versions of these procedures were the best techniques reported [12]. Nevertheless, the clinician should recognize the risks associated with these interventions, such as the development of obstructive sleep apnea and risk of hemorrhage and infection [4,7,12]. Teblick et al. also concluded that this surgical technique was associated with a lower prevalence of otitis media with effusion and lower number of ear tubes needed [19]. In the included systematic reviews, the Furlow Z-plasty technique was the most referenced form of treatment in 8 of the 13 publications evaluating invasive methods [3,4,9,14,15,17,19,20]. However, only one study was found to report statistically significant differences between the Furlow Z-plasty technique and the straight-line intravelar veloplasty closure approach, with a lower re-intervention rate of 0–11.4% vs. 0–6.7%, respectively [14]. These results should be interpreted with caution as this study was of overall low quality, namely due to the lack of independently collected data, an adequate protocol, and any considerations of the risk of bias. The remaining reviews did not present differences between several surgical techniques, but these conclusions may also have some bias, because the included studies had high clinical and methodological heterogeneity, specifically in sample size, cleft phenotype, methods used to assess speech and velopharyngeal incompetence, surgeon experience, and inclusion of syndromic patients. This heterogeneity may also affect the results of the present umbrella review and made it impossible for some SR results to be used for the meta-analysis. Overall, there appears to be no surgical technique considered to be the gold standard.

This umbrella review provides an overview of the available systematic reviews. The methodological quality of the included studies was later assessed, allowing readers to interpret the results with caution, as most studies were of low quality and did not follow a registered protocol with transparent methodology. However, this umbrella review had some limitations, such as the overall low quality of the included studies. These low-quality articles presented similar characteristics, including: lacking a clear PICO question, no list of excluded studies, no description of funding of included studies, and no assessment of the ROB effect on the statistical combination and publication bias. Additionally, most of the included studies did not include a protocol record, which may increase the occurrence of methodological flaws as well as bias. Six of the included reviews did not assess the quality of the included studies, which may also be associated with an increase in bias, as this parameter could not be used in the interpretation of the results. Furthermore, nine of the included studies did not perform a meta-analysis, which may also increase the risk of bias. Finally, the difficulties faced by investigators in conducting blind trials in the surgical field may lead to an overestimation of the treatment effects, because knowledge of the outcome may have affected the clinicians’ experiences.

Future studies should be conducted using a blind randomized controlled trial (RCT) protocol to control sources of possible bias, namely through a randomization procedure and standardization of the intervention, use of control groups, intervention timing, measurement tools, and follow-up timing. In future RCTs, authors should look to the Medical Research Council guidance for developing and evaluating surgical interventions. This guide states that the trial team must recognize all the constituent components and steps of each intervention. In this sense, it may be convenient to carry out a pilot study before the RCT study to ensure that the clinical team can systematically identify all the steps of the intervention. Despite the steep learning curve that could affect the outcome, there are some strategies that could be adopted in future studies to minimize this factor, including randomizing patients according to surgeon rather than by intervention and performing statistical tests to assess the interference of the learning curve. The aim is to improve treatment efficacy and patient quality of life whilst reducing the need for retreatments and associated costs.

## 5. Conclusions

Velopharyngeal insufficiency treatment usually comprised speech therapy and surgical intervention. Although there is no surgical technique considered to be the gold standard, the Furlow Z-plasty technique and minimal incision palatopharyngoplasty were among the best reported.

## Figures and Tables

**Table 1:** PICO question.

PICO Question	
Population	Cleft palate patients (unilateral or bilateral)
Intervention	Invasive (surgical or other medical procedures) and non-invasive (prosthetic devices, physical therapy, speech therapy) methods
Comparison	Different available interventions
Outcome	Resolution of velopharyngeal dysfunction

**Table 2:** Search keys in various databases.

Databases	Search Keys
Pubmed via Medline	(“cleft palate” [MeSH] OR “cleft palate” OR “oral cleft*” OR “orofacial cleft*”) AND (“Velopharyngeal Insufficiency” [Mesh] OR velopharyngeal OR VPI OR Palatopharyngeal Filters: systematic reviews
Web of Science	TS = (“cleft Palate*“ OR “oral cleft*” OR “orofacial cleft*” OR “Palate*, Cleft”) AND TS = (velopharyngeal OR VPI OR Palatopharyngeal) TS = (“cleft Palate*” OR “oral cleft*” OR “orofacial cleft*” OR “Palate*, Cleft”) AND TS = (velopharyngeal OR VPI OR Palatopharyngeal) AND (TS = ”Systematic review*”)
Cochrane Library	#1 MeSH descriptor: [Cleft Palate] explode all trees #2 Cleft palate #3 oral cleft* #4 orofacial cleft* #5 MeSH descriptor: [Velopharyngeal Insufficiency] explode all trees #6 velopharyngeal #7 VPI #8 Palatopharyngeal
EMBASE	(‘cleft palate’/exp OR ‘cleft palate’ OR ‘oral cleft*’ OR ‘orofacial cleft*’) AND (‘palatopharyngeal incompetence’/exp OR velopharyngeal OR palatopharyngeal OR vpi) AND ‘review’/it

**Table 3:** Non-invasive methods.

Author/Year	Design	Registration	No. of Trials and Design	Bias Analysis	Quality of Evidence	Age of Participants	Intervention	Comparison Unit	Primary Outcome	Results
Neumann et al., 2012 [13]	SR	NR	RCT (1) Single-case studies or case-series studies (5)	R	Very low (level 4)	7–50 years	(*n* = 83) Nasopharyngoscopy biofeedback (NPB)	NR	- Activation of lateral pharyngeal wall and velopharyngeal closure in articulation - Reduction of hypernasality - Nasal emission or nasal turbulence - Improvement of articulation or intelligibility in connected speech	Preliminary results show effectiveness of visual feedback by flexible nasopharyngoscopy in helping older children and adults improve their VPC during articulation, but only in combination with conventional speech therapy. No studies published measuring the effectiveness of NPB without additional treatments, such as secondary surgery or speech-language therapy. Thus, no conclusive evidence of effectiveness of NPB as a unique therapeutic method.

**Table 4:** Invasive methods.

Author/Year	Design	Registration	No. of Trials and Design	Bias Analysis	Quality of Evidence	Age of Participants	Intervention	Comparison Unit	Primary Outcome	Results
Timbang et al., 2014 [14]	SR	NR	RS (11) RCT P (1)	NR	NR	9–18 months (age at palate repair); >4 years (estimated age at speech assessment)	(*n* = 927) Repair of the cleft palate with Furlow double-opposing Z-plasty	(*n* = 1205) Repair of the cleft palate with straight-line intravelar veloplasty	- Speech (need for secondary procedures and hypernasality) - Oronasal fistula	**Furlow group:** - Need for secondary procedures to correct VPI: 0–11.4% in isolated cleft palate (ICP) and 0–6.7% in unilateral cleft lip and cleft palate (UCLP); - Hypernasality: 13–14.3% in ICP and 8.9–18.5% in UCLP; - Oronasal fistula rate: 7.87% (*p* = 0.14). **Straight-line intravelar group:** - Need for secondary procedures to correct VPI: 9.1–29.2% in ICP and 6.7–19.4% in UCLP; - Hypernasality: 11.1–20.0% in ICP and 29.1–33.3% in UCLP; - Oronasal fistula rate: 9.81% (*p* = 0.14).
Nigh et al., 2017 [11]	SR	NR	15	NR	NR	2–56 years	(*n* = 251) Autologous fat injection (combined with surgery; augmentation of soft palate alone; posterior pharyngeal wall augmentation; combined soft palate, posterior, and lateral wall augmentation)	Traditional VPI surgical treatments	- Speech quality - Rate of velopharyngeal insufficiency (RVPI) - Velopharyngeal distance by magnetic resonance imaging (MRI) - Nasometry - Nasendoscopy	In general, AFI should be reserved for patients with mild to moderate VPI (usually <50% closure gap defect or a closure defect between 0.5 and 2 cm^2^ with adequate velar mobility). Majority of studies, with one exception, required a trial of speech therapy to maximize mobility of the velum prior to AFI. Studies that included patients with VPI secondary to velocardiofacial syndrome reported satisfactory results with no major complications. Major complications were rare. Only one patient with graft hypertrophy reported obstructive sleep apnea.
Kurnik et al., 2020 [4]	SR/MA	Prospero	Retrospective cohort (10) Prospective cohort (5) Cohort (3)	R	NR	Any age	Palate re-repair: Furlow double-opposing Z-plasty, radical intravelar veloplasty (IVVP), and radical IVVP with mucosal lengthening.	NR	- Hypernasality - Nasal air emission - Additional velopharyngeal surgery - Obstructive sleep apnea	The overall incidence of achieving no consistent hypernasality following palate re-repair was 61% (95% CI: 44–75%). The incidence of achieving no hypernasality, a more stringent outcome, was 53% (95% CI: 40–65%). The incidence of less than mild hypernasality, a less stringent outcome, was 65% (95% CI: 54–75%). The incidence of no consistent nasal air emission was 78% (95% CI: 60–89%). The incidence of additional velopharyngeal surgery for persistent VPI symptoms was 21% (95% CI: 12–33%). The overall incidence of OSA following re-repair was 28% (95% CI: 13–49%). The incidence of OSA following re-repair (86%) was substantially lower than the incidence of OSA following pharyngeal flap (95%; CI: 63–96%; *p* = 0.0007). Radical IVVP had a higher incidence of achieving no consistent nasal air emission compared with Furlow DOZ (*p* = 0.0081). For the remainder of the speech outcomes there was no significant difference among techniques (*p* > 0.10). The indication for performing re-repair was not associated with the incidence of achieving no consistent hypernasality (*p* = 0.6572)
Rossell-Perry et al., 2021 [15]	SR	Prospero	10	Oxford CEBM and GRADE	Low	NR	(*n* = 503) Radical intravelar veloplasty (IVVP)	(*n* = 864) Nonradical IVV (preserving the attachment of the tendon of the tensor veli palatini muscle)	- Evaluation of speech development - Middle ear function	Definitive conclusions could not be drawn regardingthe effectiveness of radical IVV on velopharyngeal and middle ear function.
Bell et al., 2021 [7]	SR/MA	NR	29	NR	Level 3 evidence	3–75 years	(*n* = 116) Injection pharyngoplasty with (*n* = 5) GAX collagen, (*n* = 36) calcium hydroxyapatite, (*n* = 72) dextranomer and hyaluronic acid, (*n* = 3) acellular dermal matrix (Alloderm ^®^)	(*n* = 471) Injection pharyngoplasty with autologous fat	- Changes in resonance (reduction in hypernasality) - Degree of velopharyngeal closure	Functional improvements in nasality were recorded in a large proportion of patients (0.79, 95% CI: 0.75–0.82). When stratified for injection material, the proportion of patients with reduced or resolved hypernasality among those receiving synthetic injections was 0.88 (95% CI: 0.82–0.95) and 0.75 (95% CI: 0.71–0.80) for those receiving autologous fat injections (*χ*2 = 7.035, *p* = 0.008). Complete velopharyngeal gap closure post-injection was achieved at a higher frequency with injection of synthetic materials compared with autologous fat (*χ*2 = 11.270; 88% of *n* = 61/69 vs. 64% of *n* = 58/91; *p* = 0.001).
Gilleard et al., 2014 [3]	SR	NR	OS (11) CS (14) RCT (1)	NR	Methodological quality score of 6/12; Cohen kappa coefficient 0.63 (range 0.27 to 0.81)	NR	Surgery for VPI in SMCP	Z-palatoplasty; pharyngeal flap; radical velar muscle correction; island flap pushback (Millard); and pharyngeal flap	- Assessing speech outcome following surgery in SMCP - In *n* = 2, speech was evaluated from previously taken audio/video recordings (Ysunza et al., 2001; Sommerlad et al., 2004), whereas for the others, it was evaluated live	Furlow Z-plasty = 67– 97% (Chen et al., 1996; Sullivan et al., 2011), muscle correction/retropositioning = 30–33% (Sommerlad et al., 2004; Reiter et al., 2011; Sullivan et al., 2011), pharyngeal flap surgery = 32–100% (Crikelair et al., 1970; Porterfield et al., 1976; Peat et al., 1994; Isotalo et al., 2007; Sullivan et al., 2011), and sphincter pharyngoplasty 50–72% (Seagle et al., 1999; Pryor et al., 2006).
Blacam et al., 2018 [9]	SR	Pros	RCT(2) Case-control studies (3) Cross sectional studies (2) Retrospective case series (76/83, 91.5%)	Cochrane guidelines	Level IV evidence (According to the 2011 Oxford centre for evidence-based medicine criteria)	9.64 years (range 1–69.1 years)	Surgery for VPD	Pharyngeal flap; sphincter pharyngoplasty; palatoplasty; and posterior pharyngeal wall augmentation	- Speech assessment, need for further surgery, and occurrence of OSA were the outcomes of interest	Pharyngeal flap surgery was the most common procedure (64% of patients). Overall, 70.7% of patients attained normal resonance and 65.3% attained normal nasal emission. There was no notable difference in speech outcomes, need for further surgery, or occurrence of OSA across the four categories of surgery examined.
Haenssler et al., 2020 [16]	SR	NR	Retrospective reviews (11)	Risk bias was not performed	NR	NR	Buccal myomucosal flap surgical approach used for primary palatoplasty and secondary surgery for velopharyngeal insufficiency (VPI) in individuals with cleft palate	NR	- Speech and velopharyngeal competence outcomes following the buccal myomucosal flap surgical approach used for primary palatoplasty and secondary surgery for velopharyngeal insufficiency (VPI) in individuals with cleft palate	Post-surgery, normal resonance was achieved in 77.4% of patients and no nasal air emission was reported in 54.7% of patients. An improvement in velopharyngeal closure was reported in 81.8% of patients. A variety of perceptual speech assessment scales and methods for assessing velopharyngeal competence were used in the studies.
Salna et al., 2019 [17]	SR	NR	RCT (7) PS (1)	NR	low-level evidence	5.5 years	Adenoidectomy	NR	VPI following adenoid surgery	Nearly all patients showed improvement in nasal airway obstruction and snoring. The pooled risk for velopharyngeal insufficiency across all studies was 2 out of 122, which approximates to 1.6% of patients. There were very few complications.
Collins et al., 2012 [18]	SR/MA	NR	RCT (2)	*p* value of 0.10 and an I2 value of 64%	Detsky and MINORS scales The intra-class coalition coefficient was 0.977 (95% CI: 9.0–99.0%).	NR	Operative procedures for the treatment of velopharyngeal insufficiency	Pharyngeal flap or sphincter pharyngoplasty	Velopharyngeal insufficiency resolution	The forest plot of this data was produced through a random effects model analysis. The odds ratio was found to be 2.95 (95% CI: 0.66–13.23) in favour of the pharyngeal flap.
Téblick et al., 2018 [19]	SR	NR	RCT (19) prospective cohort studies (4)	NR	For level of evidence, all studies were level 2 (*n* = 3) or 3 (*n* = 20)	2 to 28 years	Cleft palate repair surgical technique	Furlow double-opposing Z-plasty; intravelar veloplasty;von Langenbeck palatoplasty; VWK, Veau-Wardill-Kilner 2-flap palatoplasty	Otitis media with effusion and disturbed speech after cleft palate repair	Four out of five studies concluded that the Furlow palatoplasty, von Langenbeck palatoplasty, VWK palatoplasty, and Sommerlad IVVP had no relevant effect on OME prevalence. Only one study reported a lower incidence of OME after the Kriens IVVP compared with the VWK palatoplasty.
Spruijt et al., 2012 [20]	SR	No		Cochrane Collaboration’s tool	Levels 2c or 4 evidence	2.4–31 years	Surgical procedure	Fat injection, Furlow, intravelar veloplasty (IVP), PF, Honig, SP, or Hynes	Determined whether a particular surgical procedure results in a greater percentage of postoperative normal resonance in patients with 22qDS and VPD	None of the interventions in current use were completely successful in correcting VPD. The low rate of normal resonance may be attributed to the short postoperative follow-up after which the full effect of speech therapy has not yet been achieved.

AFI—autologous fat injection; CI—confidence interval; CS—comparative series; DOZ—double-opposing Z-plasty; ICP—isolated cleft palate; IVVP—intravelar veloplasty; NPB—nasopharyngoscopy biofeedback; NR—not registered; OME—otitis media effusion; OS—observational series of a single procedure; OSA—obstructive sleep apnea; PS—prospective studies; R—registered; RCT—randomized controlled trial; RCT—P-randomized controlled trial prospective; RS—retrospective study; SMCP—submucous cleft palate; SR—systematic review; SR/MA—systematic review and meta-analysis; VPI—velopharyngeal insufficiency; UCLP—unilateral cleft-lip-cleft palate; VPC—velopharyngeal closure; VPD—velopharyngeal disfunction; VWK—Veau-Wardill-Kilner.

**Table 5:** Quality assessment of the included reviews, using the AMSTAR2 tool.

Author/Year	PICO	Protocol	Inclusion Criteria	Comprehensive Search	Duplicate in Selection	Duplicate in Data Extraction	List of Excluded Studies	Description of Included Studies	Assessing Risk of Bias	Funding of Included Studies	Results of Statistical Combination	ROB Effect on the Statistical Combination	ROB in the Discussion	Discussion for the Heterogeneity	Publication Bias	Author’s Funding and COF Reporting	Overall Quality
Bell et al., 2021 [7]	No	No	No	Partial Yes	Yes	No	No	Partial Yes	No	No	Yes	No	No	Yes	No	Yes	Low
Blacam et al., 2018 [9]	No	Partial Yes	No	Partial Yes	No	No	No	Partial Yes	Partial Yes	No	No	No	Yes	Yes	No	Yes	Low
Collins et al., 2012 [18]	Yes	Yes	No	Partial Yes	Yes	Yes	No	Partial Yes	Partial Yes	No	Yes	Yes	No	Yes	No	Yes	Low
Gilleard et al., 2014 [3]	No	Partial Yes	No	Partial Yes	No	Yes	No	No	Partial Yes	No	No	No	No	No	No	No	Low
Haenssler et al., 2020 [16]	No	No	No	Partial Yes	No	No	No	No	No	No	No	No	No	Yes	No	No	Low
Kurnik et al., 2020 [4]	No	Partial Yes	No	Partial Yes	Yes	Yes	No	Partial Yes	No	No	Yes	No	No	Yes	No	Yes	Low
Neumann et al., 2012 [13]	No	Partial Yes	No	Partial Yes	Yes	Yes	No	Yes	Partial Yes	No	No	No	Yes	Yes	No	No	Low
Nigh et al., 2017 [11]	No	No	No	Partial Yes	No	Yes	No	No	No	No	No	No	No	No	No	No	Low
Rossell-Perry et al., 2021 [15]	Yes	Partial Yes	No	Partial Yes	Yes	Yes	No	No	Partial Yes	No	No	No	No	Yes	No	Yes	Low
Salna et al., 2019 [17]	No	No	No	Partial Yes	Yes	Yes	No	Partial Yes	No	No	No	No	No	Yes	No	No	Low
Spruijt et al., 2012 [20]	No	No	Yes	Partial Yes	No	No	No	Partial Yes	Yes	No	Yes	No	Yes	Yes	No	Yes	Low
Téblick et al., 2018 [19]	No	No	No	Partial Yes	Yes	Yes	No	Partial Yes	Yes	No	No	No	No	No	No	No	Low
Timbang et al., 2014 [14]	No	No	No	Partial Yes	Yes	No	No	Partial Yes	No	No	Yes	No	No	Yes	No	No	Low

PICO–population, intervention, comparison, and outcome; ROB–risk of bias; COF–conflict of interests.

**Table 6:** Citation matrix for duplicate primary studies.

	Systematic Reviews												
Primary Studies	Neumann et al., 2012	Timbang et al., 2014	Nigh et al., 2017	Kurnik et al., 2020	Rossell-Perry et al., 2021	Bell et al., 2021	Gilleard et al., 2014	Blacam et al., 2018	Haenssler et al., 2020	Salna et al., 2019	Collins et al., 2012 (*n* = 2)	Téblick et al., 2018	Spruijt et al., 2012
Abdel-Aziz et al., 2007							x	x					
Antonelli et al., 2011					x							x	
Argamaso et al., 1994								x					x
Arneja et al., 2008								x					x
Boneti et al., 2015			x			x		x					
Brandao et al., 2011								x					x
Brigger et al., 2010						x		x					
Cantarella et al., 2011			x			x		x					
Cao et al., 2013			x					x					
Chen et al., 1994				x				x					
Chen et al., 1996							x	x					
DÁndrea et al., 2018					x							x	
Dejonckere and van Wijngaarden et al., 2001			x			x							
Deren et al., 2005				x				x					
Doucet et al., 2013					x							x	
Filip et al., 2013			x			x		x					
Filip et al., 2011			x			x							
Guerrerosantos et al., 2004			x			x							
Klotz et al., 2001			x			x							
Lau et al., 2013			x			x		x					
Leboulanger et al., 2011			x			x		x					
Leuchter et al., 2010			x			x							x
Logjes et al., 2017				x					x				
Mazzola et al., 2015			x			x							
Mehendale et al., 2004								x					x
Milczuk et al., 2007								x					x
Nakamura et al., 2003				x				x					
Park et al., 2000							x	x					
Pensler et al., 1988							x	x					
Piotet et al., 2015			x			x		x					
Robertson et al., 2008				x					x				
Rouillon et al., 2009								x					x
Sie et al., 1998								x					x
Spruijt et al., 2011								x					x
Widdershoven et al., 2008								x					x
Yu et al., 2014		x										x	

Two duplicate citations in primary studies (yellow color); three duplicate citations in primary studies (orange color).

## Data Availability

The data presented in this study are available on request from the corresponding author.
